# Accuracy of Healthcare Professionals’ Estimations of Health Literacy and Numeracy of Patients Visiting Metabolic Bariatric Surgery Clinic

**DOI:** 10.1007/s11695-024-07379-y

**Published:** 2024-07-04

**Authors:** Calisha Allen, Lubnaa Ghoora, Rajashree Murki, Chad Byworth, Sarah Beale, Akifah Mojadady, Jameela Nagri, Chetan Parmar

**Affiliations:** 1 Institute of Health Informatics, University College London, London, NW1 2DA UK; 2 The Royal Free London NHS Foundation Trust, London, NW3 2QG UK; 3 Department of Surgery, Whittington Hospital, London, N19 5NF UK; 4 Apollo Hospitals Educational and Research Foundation, New Delhi, India; 5 Department of Targeted Intervention, University College London, London, UK

**Keywords:** Health literacy, Health numeracy, Obesity, Bariatric clinic

## Abstract

**Introduction:**

To effectively support patients through their weight loss journey, it is vital that healthcare professionals (HCPs) understand the health literacy skills of their patients and communicate in a way that meets these needs. This is the first study looking at the accuracy of HCPs’ estimations of their patients’ health literacy and numeracy attending a metabolic bariatric surgery (MBS) clinic.

**Method:**

A cross-sectional study was completed at a tertiary-level MBS clinic in London. Patients completed a demographic questionnaire and a validated measure of health literacy and numeracy, the Medical Term Recognition Test (METER) and General Health Numeracy Test–Short Form (GHNT-6), respectively. HCPs provided estimations of their patient’s health literacy and numeracy based on each questionnaire’s scoring categories.

**Results:**

Data was collected for 31 patients. A 80.6% of patients had functional health literacy based on METER. HCPs estimated patients’ health literacy correctly 61.1% of the time; inter-rater agreement was poor (ICC = 0.14; 95% CI =  − 0.19, 0.443; *p* = 0.202).

A total of 22.6% of patients scored 0 out of 6 on GHNT-6. HCPs estimated health numeracy correctly 13.9% of the time and were more likely to overestimate than underestimate health numeracy. Inter-rater agreement for health numeracy was poor (ICC =  − 0.2; 95% CI =  − 0.49, 0.14; *p* = 0.878).

**Conclusion:**

There is poor agreement between HCPs’ perception of their patients’ health literacy and numeracy and their assessed ability. HCPs’ understanding of their patient’s health literacy and numeracy skills is vital in ensuring HCPs can support patients through the challenging bariatric surgical pathway, consenting process and post-operative course.

**Graphical Abstract:**

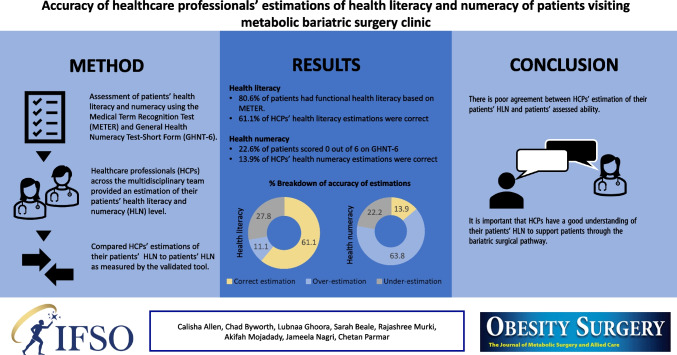

## Introduction

Literacy skills refer to the ability to read, understand and utilise information and the ability to communicate ideas through both verbal and written communication [1]. Numeracy refers to mathematical skills and the ability to apply these to different situations [2]. Health literacy and numeracy refer to an individual’s ability to use their literacy and numeracy skills in the health environment [3]. This can include the use of these skills in formal care environments such as in a clinic or on the ward, as well as in day-to-day life through self-care. Health literacy and numeracy are known to be associated with a range of demographic factors including age, education level, socio-economic status, perception of one’s own health status and Internet access [4].

Health literacy and numeracy can affect a patient’s ability to correctly administer medication, take part in preventative care and read and utilise health information [3, 5, 6]. A patient’s health literacy and numeracy can be measured using validated health literacy tools, with a range of tools available to measure general and disease-specific health literacy and numeracy skills. Health literacy and health numeracy whilst associated should be recognised as separate skills. Lower health literacy is associated with lower health numeracy; however, even those with adequate health literacy have been shown to achieve low scores on health numeracy assessments[7]. Previous studies have demonstrated that 42% of working-age adults do not have the health literacy skills to utilise everyday health information, rising to 61% for health numeracy skills [8]. It is estimated that 31.7% (95% CI 24.7–39.2%) of surgical patients have limited health literacy [9].

For patients attending bariatric surgical clinics, health literacy and numeracy levels are relevant as they are known to affect weight loss after surgery and the ability of patients to maintain adequate nutrition post-surgery and are associated with an increased risk of post-surgical complications [10, 11]. Research demonstrates that health literacy is also known to affect the consultation and patients’ ability to take part in shared decision-making. Similarly, poor health literacy can affect comprehension during the consenting process for surgical interventions [11, 12]. However, where lower health literacy is identified, decision-making aids can be used to support patients, and steps such as teach-back can be taken within the consultation to improve and ensure patient understanding [13]. Additionally, simple modifications to written and verbal communication, using different language and pictures or demonstrations, can support increased understanding in patients with low health literacy [14].

Previous research has found that healthcare professionals (HCPs) have poor accuracy in estimating their patient’s health literacy and numeracy. Healthcare professionals overestimated health literacy 22–58% of the time and underestimated health literacy 5–29% of the time, with studies conducted in a range of healthcare settings including primary and secondary care [15]. To the authors’ knowledge, this study is the first to focus on health literacy and numeracy in patients attending a MBS clinic and to investigate the accuracy of estimations from a range of members of the multi-disciplinary team (MDT) working within the clinic.

## Ethics Application and Approval

This study was approved by the Health Research Authority and Health and Care Research Wales (22/NS/0053), and ethics approval was given by the North of Scotland Research Ethics Committee.

## Methods

A cross-sectional study was completed at a tertiary-level bariatric clinic at the Whittington Hospital, London. Participants were voluntarily recruited from the waiting room of the MBS clinic. Recruitment days occurred over 8 months across 2022–2023, and all patients meeting the inclusion criteria were approached. The inclusion criteria for patients stated that they must be over 18 years old, be able to consent in English and not need an interpreter for their consultation, although this will not exclude patients who do not speak English as their first language.

Participants who volunteered to participate were given a paper questionnaire. Informed consent was obtained from all individual participants included in the study. The questionnaire looked at a range of demographic factors mirroring a selection of questions from the 2021 Office for National Statistics (ONS) census. Health literacy was measured using the Medical Term Recognition Test (METER) [16]. METER is a self-administered paper questionnaire designed with a mix of real words and nonwords, and participants are asked to identify the words they know to be real words. Participants could score a max of 40 marks, and there was no negative marking [16]. Health numeracy was measured through the General Health Numeracy Test–Short Form (GHNT-6), a self-administered paper questionnaire. There were 6 questions, with a maximum score of 6 and no negative marking [17]. There was no time restriction for the participants to fill out the questionnaire.

All registered HCPs in the clinic were eligible to take part, and this included nurse specialists, consultant surgeons, surgical registrars and dieticians. HCPs who were happy to take part were consented, shown the questionnaires that would be filled out by the patients and instructed not to change their consultation. At the end of clinic, HCPs were asked to give estimations of the health literacy and numeracy scores for the patients they saw. HCPs were asked to estimate health literacy using the categorical groups of low, marginal and functional health literacy which aligned with scores of 0–20, 21–34 and 35–40. HCPs were asked to estimate health numeracy by estimating a score out of 6.

### Data Analysis

All estimations and patient scores were recorded on paper and transcribed by the researchers into an Excel spreadsheet. This was imported and analysed using R 4.2.3.

Demographic characteristics were analysed using descriptive statistics. The accuracy of HCP’s estimations is described descriptively through percentages and statistically through the intraclass correlation coefficient (ICC). ICC and 95% confidence intervals were completed on R using package “irr”, based on single ratings, using a one-way random effects model. The results will be interpreted using the suggestions from Cicchetti (1994) with < 0.4 for poor agreement, 0.4–0.59 for fair agreement, 0.6–0.74 for good and 0.75–1.00 for excellent [18].

In addition to the overall score, questions 1 and 4 in each GHNT-6 questionnaire were recorded individually to ascertain the proportion of patients who correctly answered these two questions.

## Results

A total of 31 patients were recruited from an outpatient MBS clinic. The mean age of patient participants was 41.9 years (standard deviation (SD) 12.1), 83.9% of patient participants were female, and the mean body mass index (BMI) was 41.8 kg/m^2^ (SD 9.4). Table 1 further details demographic factors.

**Table 1:** Participant demographics (*n* = 31)

	**Count (%)**	
Age^a^		
20–30	6 (20)	
31–40	9 (30)	
41–50	6 (20)	
51–60	6 (20)	
≥ 61	3 (10)	
Age (median, years)	39 years	
Gender		
Man	5 (16.1)	
Woman	26 (83.9)	
Ethnicity^a^		
White	14 (46.7)	
Black, Black British, Caribbean or African	8 (26.7)	
Mixed or multiple ethnic groups	4 (13.3)	
Other ethnic group	3 (10)	
Asian or Asian British	1 (3.3)	
First language		
English	25 (80.6)	
Other languages	6 (19.4)	
Place of Birth		
Born in the UK	23 (74.2)	
Born outside of the UK	8 (25.8)	
Highest qualification		
≤ Level 3^b^	16 (51.6)	
≥ Level 4^b^	13 (41.9)	
Other	2 (6.5)	
Home internet access^a^		
Access to the internet at home	29 (96.7)	
No access to the internet at home	1 (3.3)	
	**Mean**	**SD**
BMI	41.8	9.4

^a^Data for 1 participant is missing ^b^In the UK, qualifications are classified based on their level of difficulty from entry level 1 to level 8. Level 3 qualifications include A levels and the international baccalaureate that students may leave school with commonly at aged 18. Level 4 qualifications include a certificate of higher education or a higher apprenticeship. An undergraduate degree is classified as level 6[19]

All participants completed the health literacy questionnaire with 80.6% (*n* = 25) having functional health literacy and 6.5% (*n* = 2) having low health literacy. HCPs’ estimations were provided by consultants, registrars, specialist nurses and dieticians, with a total of 36 health literacy estimations provided. A total of 61.1% (*n* = 22) of estimations were correct. HCPs were more likely to underestimate than overestimate health literacy, when they estimated incorrectly. Underestimations accounted for 71.4% (*n* = 10) of incorrect estimations. Agreement analysed through ICC for health literacy showed poor agreement between HCPs’ estimations and measured health literacy through METER (ICC = 0.14; 95% CI =  − 0.19, 0.443; *p* = 0.202).

The health numeracy questionnaire GHNT-6 was completed by all participants. A total of 22.6% (*n* = 7) of participants scored 0 out of 6, and only 3.2% (*n* = 1) of participants answered all questions correctly. In the population, GHNT-6 was validated, the average score was 42% (2–3 out of 6); in this study population, 38.8% (*n* = 12) scored below this, and the average score was 41.7% [17]. The breakdown of scores can be seen in Table 2.

**Table 2:** GHNT-6 scores, participants (*n* = 31)

Raw score GHNT-6	*n* (%)
0	7 (22.6)
1	5 (16.1)
2	4 (12.9)
3	4 (12.9)
4	3 (9.7)
5	7 (22.6)
6	1 (3.2)

**Fig. 1:**
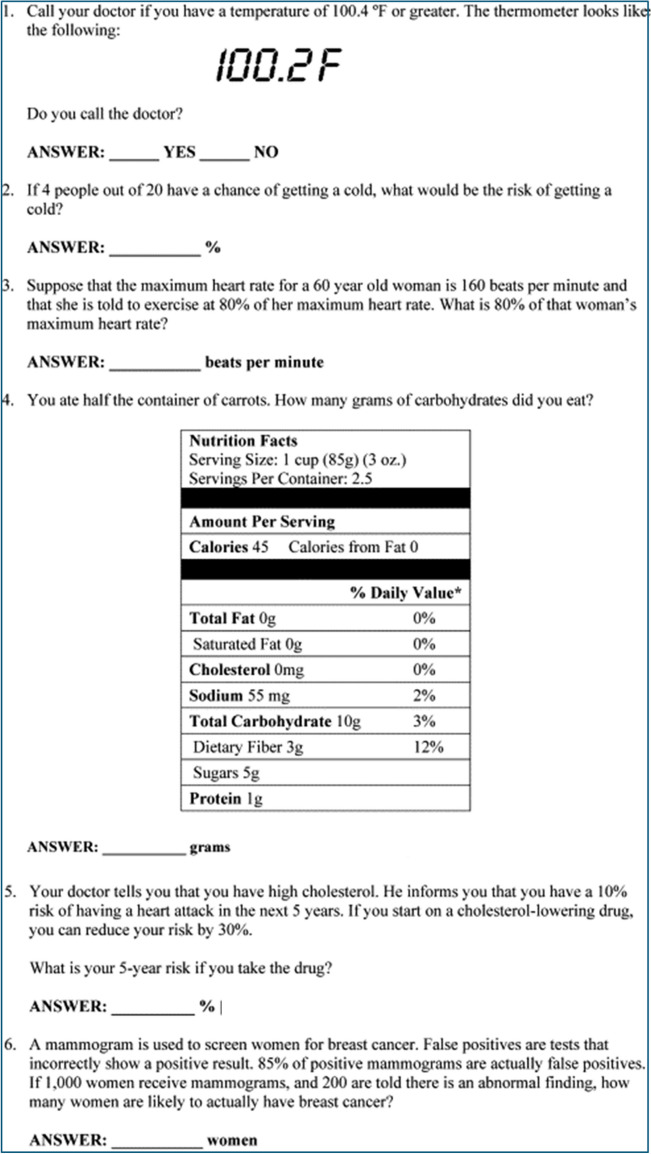
Questions in GHNT-6 [17]

Question 1 asked patient participants to decide if the thermometer reading was greater than 100.4 °F (Fig. 1). This question was answered correctly by 74.2% (*n* = 23) of participants. In question 4, participants were asked to calculate carbohydrates from reading a food label. This was answered correctly by 19.4% (*n* = 6) of patient participants (Fig. 1).

HCPs provided 36 estimations of health numeracy, and 13.9% (*n* = 5) of estimations were correct. Where estimations were incorrect, HCPs were more likely to overestimate than underestimate a patient’s health numeracy. Overestimations accounted for 63.9% (*n* = 23) of all estimations and 74.2% of all incorrect estimations. Inter-rater agreement was tested through ICC and demonstrated poor agreement between HCP’s estimations and a patient’s health numeracy as measured by GHNT-6 (ICC =  − 0.2; 95% CI =  − 0.49, 0.14; *p* = 0.878).

There was a weak association between health literacy and health numeracy (Spearman’s rho = 0.37, *p* < 0.05), as health literacy increased, health numeracy scores increased. The average health numeracy score for those with low health literacy who scored 0–20 on METER was 0.5, rising to 2.7 for those with functional health literacy, those scoring 35–40 on METER.

## Discussion

This study explored the accuracy of healthcare professionals’ estimation of patients’ health literacy and numeracy as compared to patients’ health literacy and numeracy as measured by a validated questionnaire. The study found that 61.1% of HCPs’ health literacy estimations were correct, and where they were incorrect, HCPs were more likely to underestimate than overestimate their patient’s health literacy. In contrast, HCPs were most likely to overestimate health numeracy, accounting for 63.8% of estimations and correctly estimated health numeracy only 13.9% of the time.

Previous studies, mostly conducted in the USA, using composite measures of health literacy and numeracy such as the Test of Functional Health Literacy in Adults (TOFHLA), found that HCPs correctly estimated health literacy 13–61% of the time. These studies have looked at individual healthcare professional groups with the vast majority of studies being completed in doctors [15]. This study looked at the team as an MDT and included all types of HCPs conducting a consultation with the patient. Ours is the first study of its kind to look at the population attending a MBS clinic and including the full breadth of the MDT, including dieticians and nurses and collecting health numeracy data separately to allow for a separate review of health numeracy skills from health literacy.

The patient cohort in this study had a mean age of 41.9 (SD 12.1) years compared to the London regional mean of 36.9 (SD 21.6). The study population disproportionately identified as women as compared to the London region (83.9% vs. 47.6%) and had a greater proportion of individuals with a level 4 qualification or greater (41.9 vs. 33.9%), such as a university degree or professional qualification. More women undergo MBS and that explains the reason why our study population had higher numbers of females.

In this study population, the majority of patients had functional health literacy, however, patients’ scores in health numeracy varied, with just over a third scoring 1 or less. This reflects wider findings on general literacy and numeracy skills by the Organisation for Economic Co-operation and Development (OECD) which demonstrated that in England, a greater proportion of the population has poor numeracy skills than literacy skills [20].

Question 4 of the health numeracy questionnaire tested the patient participants’ ability to read and use a food label and required a range of skills to complete it. Based on the adult numeracy core curriculum, participants were required to use entry level 3 skills to divide a decimal of 2.5 to 1.25 to calculate the number of servings and level 1 skills to multiply a decimal by 10 [21]. The 2011 Skills For Life survey found that only 50.8% of adults aged 16–65 had the basic numeracy skills of level 1 or greater to complete the numeracy skills in this question [22]. In addition, as a worded problem, the question had linguistic demands, and there was increased complexity due to the presence of extraneous information [23]. This question was only answered correctly by 19.4% of participants.

This study demonstrated two key issues to be considered by the reader. Firstly, the poor agreement between HCPs’ perceptions of patients’ health literacy and numeracy is important in considering how HCPs share information with patients and how we recognise when patients need additional support to be part of shared decision-making and follow treatment plans. This is particularly important in this population who are consenting to elective surgery and who will need to be able to successfully follow advice post-surgery to ensure positive outcomes and avoid complications.

Secondly, the level of health numeracy skills in the population may be unexpected to the reader, and it is particularly of note that even within the population attending a MBS clinic, less than a fifth have the skills to interpret a food label. The study population was more qualified than the general population; however, a large proportion were not able to apply everyday health numeracy skills such as reading a thermometer or food label. When consulting with patients, HCPs should be mindful of the health numeracy skill required to take on new information such as percentages when discussing risk or fractions and decimals when discussing dietary advice. These become particularly important as patients undergoing MBS will have discussions related to the proportions of proteins, carbohydrates and fats in the diet they need to follow.

Further research is required to demonstrate the health literacy and numeracy level of patients undergoing MBS in a larger sample size and across multiple centres. Further work should also be undertaken to understand the health numeracy demands along the patient journey pre- and post-op and describe opportunities to build patients’ health numeracy skills and ensure that information is accessible at all skill levels.

There are several limitations to this study. The sample is small and convenient, with patients and HCPs from a single centre. METER was validated in the USA, and as a result, some words in it such as ‘anemia’ and ‘Fam’ would have had different classifications in the UK; participants were informed at the beginning of the questionnaire that it was written in the USA. Patient participants also fed back that the format of METER was particularly difficult if you had dyslexia and so may not accurately measure your health literacy level. METER only tested reading skills and did not test oral health literacy or the ability to understand and act on advice. GHNT-6 did not test all areas of numeracy such as skills around measures of volume, weight and length which are key skills when following advice around diet and weight loss.

There is no clear evidence-based solution on how to assess health literacy and numeracy outside of research in clinical practice. Further, the use of health literacy and numeracy assessment tools outside of research in the clinical setting is complicated by the feasibility of testing patients, the possibility of causing shame and embarrassment for patients and the challenge of selecting a tool, given the large number of health literacy tools that are available [24]. Existing health literacy and numeracy tools have been used in clinical settings, but users should be aware of their limitations and continue to take universal health literacy precautions with all patients [25]. The hope of this study is to raise awareness of this issue and encourage more research in this area in the future for a better understanding of our patients.

This study demonstrates that HCPs’ estimations alone are likely inadequate to identify patients’ health literacy and numeracy needs. HCPs may therefore consider using universal health literacy precautions to support information being accessible to all patients due to the difficulties of identifying patients without functional health literacy and numeracy skills. Broadly, we can look at this in the consultations and education programmes, in our written communications and in supporting self-care. In the consultation and education programmes, we can support patients’ health literacy and numeracy by using the teach-back method; here, you ask patients to explain in their own words what they have understood. In written communication, including leaflets and letters, we can support patients by using everyday language, using images, minimising medical jargon and acronyms and explaining them when they have to be used. Finally, we can support self-care by encouraging questions, using action plan forms with patients to guide and record the management plan and making prescription easier to follow, e.g. “Take 1 tablet by mouth in the morning and in the evening” instead of “Take 1 tablet by mouth two times per day” [26]. When applicable video materials can be used as an educational tool and can be particularly helpful for explaining self-care activities. Finally, patient and public involvement is an important way to gain feedback on existing and changes to educational materials [26].

## Conclusion

Health literacy and numeracy skills play an important role in supporting patients’ pre- and successful post-operative courses. This study found that whilst the majority of patients had a functional health literacy score, just over a third scored 1 or less on the health numeracy questionnaire. There was poor agreement between HCPs’ perception of their patients’ health literacy and numeracy and their assessed ability. HCPs’ understanding of health literacy and numeracy skills is vital in ensuring that they can provide patients with information in a tailored way to support informed consent, shared decision-making and improved self-care during the post-operative journey. Taking universal health literacy precautions with all patients will support access to information given the difficulty in identifying patients facing health literacy and numeracy challenges.

## Data Availability

The data is not publicly available to maintain the privacy participants, but further anonymised aggregated data points are available from the corresponding author (CA) on reasonable request.
